# Epineurial Window Is More Efficient in Attracting Axons than Simple Coaptation in a Sutureless (Cyanoacrylate-Bound) Model of End-to-Side Nerve Repair in the Rat Upper Limb: Functional and Morphometric Evidences and Review of the Literature

**DOI:** 10.1371/journal.pone.0148443

**Published:** 2016-02-12

**Authors:** Igor Papalia, Ludovico Magaudda, Maria Righi, Giulia Ronchi, Nicoletta Viano, Stefano Geuna, Michele Rosario Colonna

**Affiliations:** 1 Department of Biomedical Sciences and Morphological and Functional Images, University of Messina, Messina, Italy; 2 Department of Clinical and Biological Sciences, University of Torino, Orbassano (Torino), Italy; 3 Neuroscience Institute of the “Cavalieri Ottolenghi” Foundation (NICO), University of Torino, Orbassano (Torino), Italy; 4 Department of Experimental and Clinical Surgical and Medical Specialties, University of Messina, Messina, Italy; University of Szeged, HUNGARY

## Abstract

End-to-side nerve coaptation brings regenerating axons from the donor to the recipient nerve. Several techniques have been used to perform coaptation: microsurgical sutures with and without opening a window into the epi(peri)neurial connective tissue; among these, window techniques have been proven more effective in inducing axonal regeneration. The authors developed a sutureless model of end-to-side coaptation in the rat upper limb. In 19 adult Wistar rats, the median and the ulnar nerves of the left arm were approached from the axillary region, the median nerve transected and the proximal stump sutured to the pectoral muscle to prevent regeneration. Animals were then randomly divided in two experimental groups (7 animals each, 5 animals acting as control): Group 1: the distal stump of the transected median nerve was fixed to the ulnar nerve by applying cyanoacrylate solution; Group 2: a small epineurial window was opened into the epineurium of the ulnar nerve, caring to avoid damage to the nerve fibres; the distal stump of the transected median nerve was then fixed to the ulnar nerve by applying cyanoacrylate solution. The grasping test for functional evaluation was repeated every 10–11 weeks starting from week-15, up to the sacrifice (week 36). At week 36, the animals were sacrificed and the regenerated nerves harvested and processed for morphological investigations (high-resolution light microscopy as well as stereological and morphometrical analysis). This study shows that a) cyanoacrylate in end-to-side coaptation produces scarless axon regeneration without toxic effects; b) axonal regeneration and myelination occur even without opening an epineurial window, but c) the window is related to a larger number of regenerating fibres, especially myelinated and mature, and better functional outcomes.

## Introduction

A number of experimental [1,2,3,4,5,6,7,8,9,10,11,12,13,14,15,16,17] as well as clinical [18,19,20,21,22,23,24,25,26,27,28,29,30,31,32,33,34,35,36,37] studies have shown that end-to-side nerve coaptation is able to induce collateral sprouting from donor nerve’s axons, allowing for significant repopulation of the distal nerve stump.

The injury to the donor nerve due to microsurgical suture in end-to-side coaptation, seems to be the starter of axonal growth and reinnervation in distal stump of receiving damaged nerve. However, it has been observed that this procedure results in the loss (“escape”) of nerve fibres from the donor nerve, acting as a restricting factor in the surgeon’s mind when choosing the technique for nerve repair [38,39,40,41,42].

In this study we evaluate the possibility that this kind of reinnervation may be obtained without microsurgical procedure and, consequently, without the loss of nerve fibres in the donor nerve.

In an experimental model the receiving (cut) median nerve was coapted to the donor healthy ulnar nerve, by means of only adhesive biocompatible substance, butyl 2-cyanoacrylate.

Cyanoacrylates are a group of substances well-known and tested for their gluing properties and to date available for clinical use [43,44,45,46,47,48]. Restrictions to their use in nerve coaptation have been registered by some authors [43,48], who detected that uncontrolled contamination by the gluing agent of the coapted nerve surfaces could produce local inflammation and a scar wall stopping nerve fibre regeneration. However, their findings have been questioned by other studies, demonstrating that a transient inflammatory effect produced by cyanoacrylate in the coaptation zone is capable to stimulate local ingrowth of Schwann cells and connective tissue, creating the way for axonal sprouting [44,45,46,47].

The aim of this study was thus 1) to develop a sutureless, less traumatic, simple and fast technique for coaptation of the distal stump of a dissected nerve onto a nearby health donor nerve with end-to-side reinnervation model and 2) to prove that the growth of axons is possible only through the biological events associated with the tropism of the receiving damaged nerve and the corresponding target organ.

The chosen experimental model was the rat upper limb, which allows a detailed analysis on functional recovery by grasping test [49,50] followed by morphological evaluation of donor and receiving nerves [51,52].

## Materials and Methods

### Surgery

For this study, 19 adult female Wistar rats, weighing approximately 200g, were utilized. Experimental surgery was carried out at the microsurgical laboratory of the Ecole de Chirurgie in Paris (Institutional license from the “Direction départementale de la protection des populations”, DDPP number C-75-05-23) according to the French law on experimental animal research (law no. 87–848, October 19, 1987). All the surgeries were carried out in the period between January 2012 and March 2013 by expert surgeons certified by the “Service protection et santé animals de le Ministère de l’Agriculture”.

All animals were housed in plastic cages with a 12/12 light/dark cycle, and water and food were available *ad libitum*. Adequate and standard measures were taken to minimize pain and discomfort taking into account human endpoints for animal suffering and distress.

After deep anesthesia induced with ketamine (40 mg/250g) and cloropromazine (3.75 mg/250g), the median and the ulnar nerves of the left upper limb were approached under operating microscope magnification (ZEISS OPMI 7, magnification 0.4/0.63/1.0/1.6/2.5) from the axillary region and carefully exposed. A 10-mm long segment of the median nerve was dissected and cut, and the proximal nerve stump was sutured to the pectoral muscle to prevent regeneration. Animals were then randomly divided in two experimental groups:

1. Group 1 (N-butyl-2-cyanoacrylate w/o epineurial window): the distal stump of the transected median nerve was fixed to the ulnar nerve by applying N-butyl-2-cyanoacrylate solution (7 animals);
2. Group 2 (N-butyl-2-cyanoacrylate with epineurial window): a small epineurial window was opened into the epineurium of the ulnar nerve, trying to avoid damage to the nerve fibres below; the distal stump of the transected median nerve was then fixed to the ulnar nerve by applying N-butyl-2-cyanoacrylate solution (7 animals).

The skin was then sutured and the animals were allowed to recover.

Five animals were used as un-injured controls (i.e. without sham operation).

### Functional assessment

Functional evaluation of median nerve recovery was assessed by the grasping test, as previously described [50]. Briefly, this test consists of holding the rat by its tail and lowering the animal towards the device. Then, when the animal grips the grid, it is pulled upward until it loses it. The balance records the maximum weight that the animal is able to hold up before losing the grip. Animals were tested every 10–11 weeks starting from week-15, up to the sacrifice (week 36); each animal was tested three times and the average value was recorded. The day before surgery, the function of the left median nerve was assessed to have the control values.

### Morphology, stereology and morphometry

36 weeks after surgery, animals were subjected to deep anesthesia (ketamine, 40 mg/250g and cloropromazine, 3.75 mg/250g) and the median nerve was approached. From each animal, the regenerated nerve was harvested. Animals were then euthanized with overdose of anesthetic and animal death was confirmed by exsanguination (abdominal aorta resection).

Nerve specimens were fixed by immediate immersion in 2.5% glutaraldehyde in 0.1 M PBS pH 7.4 for up to 6 h at 4°C, washed in Sorensen phosphate buffer 0.1 M (pH 7.4) with 1.5% sucrose, and post-fixed in 2% osmium tetroxide for 2 h. Samples were then carefully dehydrated in passages in ethanol from 30% to 100%, cleared in propylene oxide and embedded in Glauerts’ embedding mixture of resins consisting in equal parts of Araldite M and Araldite Harter, HY 964 (Merck, Darmstadt, Germany), containing 0.5% of the plasticizer dibutylphthalate and 1–2% of the accelerator 964, DY 064 (Merck, Darmstadt, Germany).

For high-resolution light microscopy, semi-thin transverse sections (2.5 μm thick) were cut starting from the distal stump of each nerve specimen, using an Ultracut UCT ultramicrotome (Leica Microsystems, Wetzlar, Germany) and stained with 1% toluidine blue.

For stereological and morphometrical analysis, design-based quantitative morphology was performed [53,54]. One toluidine-blue section was randomly selected and the cross-sectional area of the whole nerve section was measured. On the same image, 10–12 sampling fields were selected using a systematic random sampling protocol [53,55,56]. In each sampling field, the "edge effect" was avoided by employing a two-dimensional dissector method which is based on counting the "tops" of the myelinated fibers. The total number of myelinated fibers, as well as different size parameters (fiber and axon diameter and myelin thickness) were then calculated.

### Statistical analysis

Quantitative data are presented as mean + Standard Error. All data were statistically analyzed using one-way analysis of variance (SPSS software).

## Results

### Functional assessment

**Fig 1:**
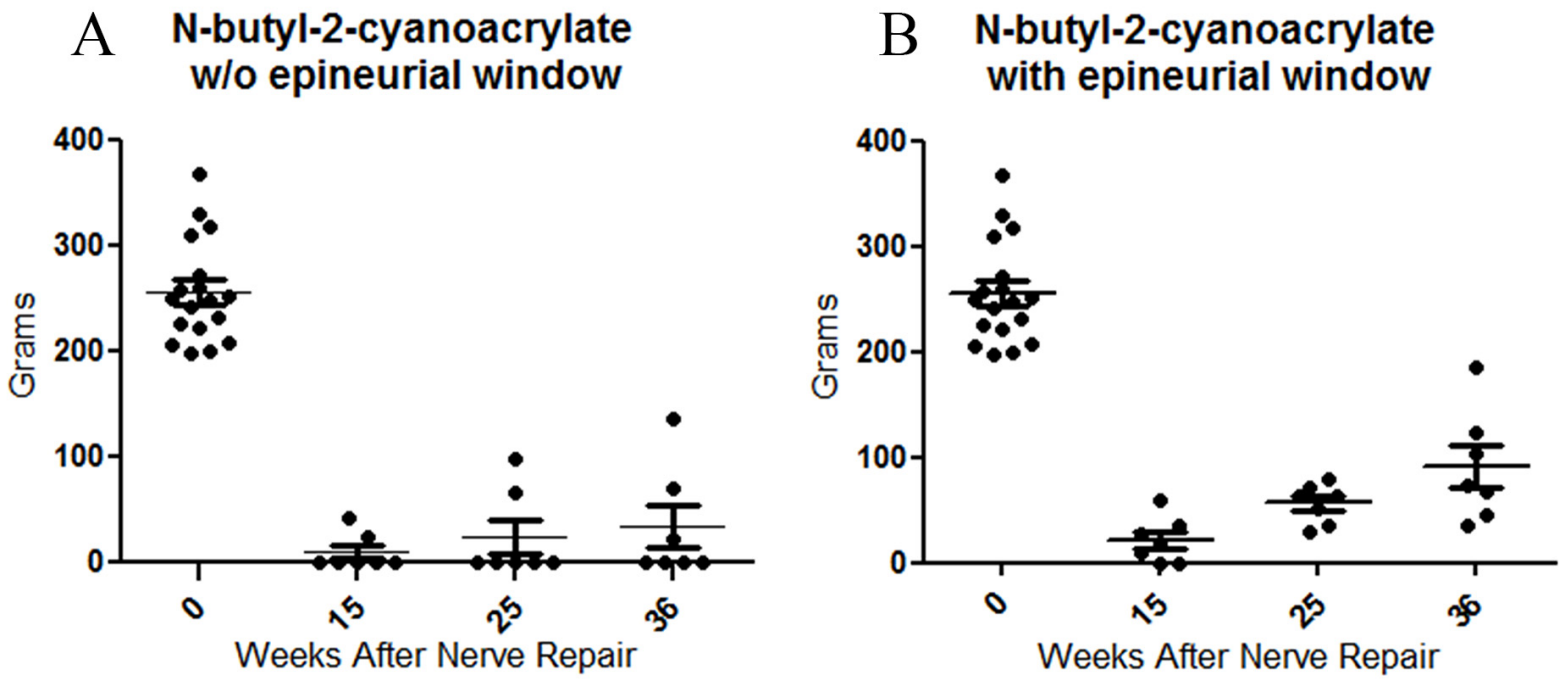
Performance of rats in the grasping test following end-to-side neurorrhaphy. A: N-butyl-2-cyanoacrylate w/o epineurial window group (group 1); B: N-butyl-2-cyanoacrylate with epineurial window group (group 2). A predominately number of animals of group 2 has recovered motor function (five animals after 15 weeks and all the seven animals after 36 weeks), compared to group 1 (only two animals recovered motor function activity after 15 weeks, and three animals after 36 weeks). Data are presented as scatterplots showing individual animal values with integrated mean and variance values.

To investigate whether N-butyl-2-cyanoacrylate solution and the presence or not of the epineurial window can interfere with the regeneration, we first evaluated motor functional recovery after end-to-side coaptation, obtained at the time of the first evaluation (pre-operative) and after 15, 25 and 36 weeks from the repair. Results are presented in Fig 1. 15 weeks after nerve repair, only two animals of Group 1 (N-butyl-2-cyanoacrylate w/o epineurial window) started to recover motor function (Fig 1A), whereas in Group 2 (N-butyl-2-cyanoacrylate with epineurial window) already five animals reached this result (Fig 1B). This discrepancy between the two experimental groups is more detectable after 25 and 36 weeks: all the animals of group 2, but only three animals of Group 1, recovered motor function at the end of the experiment (36 weeks). The remaining four animals of Group 1 did not recovered active digit flexion even 36 weeks after nerve repair. However, both experimental groups were statistically different (p ≤ 0.05) compared to control, also after 36 weeks.

### Morphological analysis

**Fig 2:**
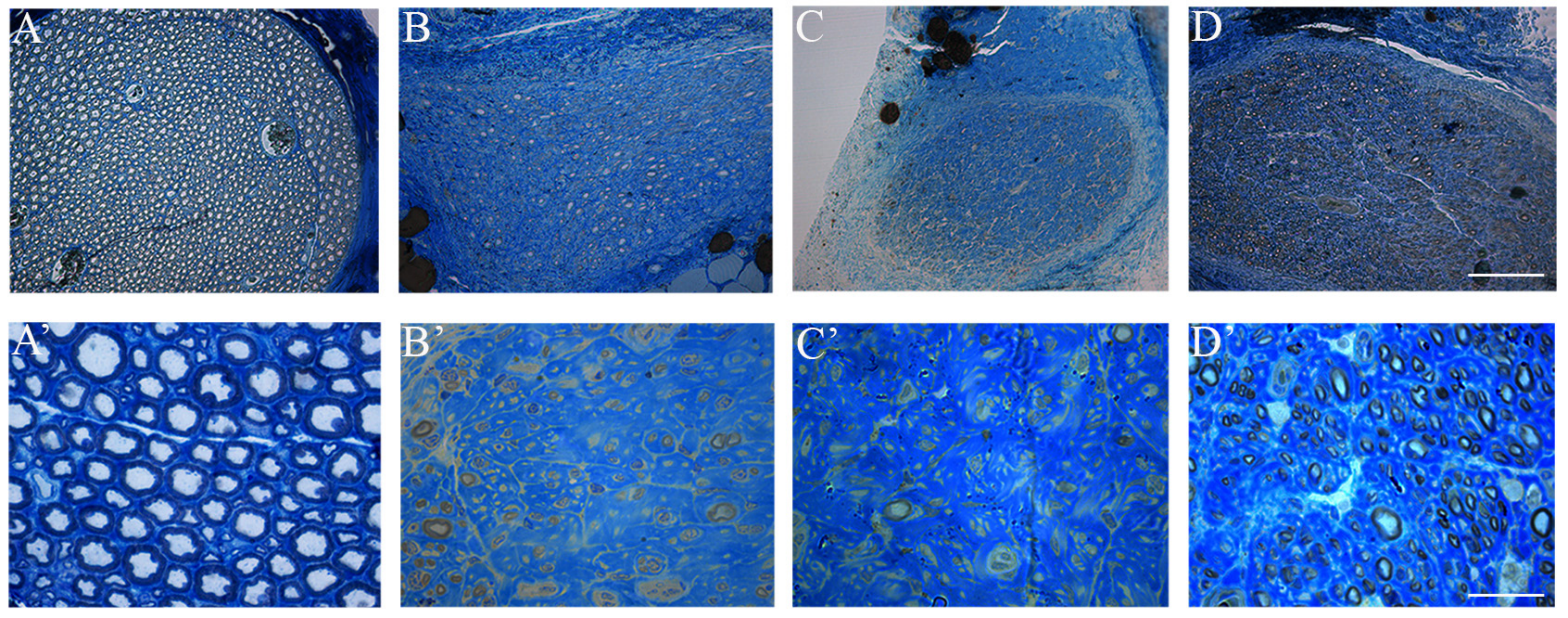
High resolution light microscopic images of a control median nerve (A-A’) and median nerves 36 weeks after end-to-side coaptation. B-B’: N-butyl-2-cyanoacrylate w/o epineurial window group, animal with functional recovery; C-C’: N-butyl-2-cyanoacrylate w/o epineurial window group, animal without functional recovery; D-D’: N-butyl-2-cyanoacrylate with epineurial window group. Both experimental groups show regenerating fibres, but animals which did not recover functional activity of Group 1 (C-C’) show smaller nerve cross sectional area with fewer and smaller fibres compared to both Group 1_with functional recovery (B-B’) and Group 2 (D-D’). Bars: A-D: 100 μm; A’D’: 10 μm.

We compared semi-thin sections of the regenerated nerves harvested 36 weeks after end-to-side coaptation. In Fig 2, representative images taken from the distal part of the median nerve are shown. Since Group 1 (N-butyl-2-cyanoacrylate w/o epineurial window) showed only a partial functional recovery (some animals recovered and some animals did not), we displayed representative pictures of both conditions (Fig 2B–2B’, Group 1 with functional recovery; 2C–2C’, Group 1 without functional recovery).

Low power images of whole cross-section of regenerated nerves showed that, in accordance with functional results, animals belonging to Group 1 that did not recovered functional activity (Fig 2C), have a smaller cross-sectional area compared to animals of Group 1 which recovered functional activity (Fig 2B) and to animals of Group 2 (N-butyl-2-cyanoacrylate with epineurial window) (Fig 2D).

Moreover, high magnification pictures showed that regenerating fibres are present in all experimental group, but animals belonging to Group 1 without functional recovery (Fig 2C’), have fewer, smaller and with thinner myelin thickness fibres compared to animals of Group 1 which recovered functional activity (Fig 2B’) and to animals of Group 2 (Fig 2D’). Among Group 1, animals with functional recovery showed regenerated fibres comparable to those of animals belonging to Group 2 from a morphological point of view.

### Stereological and morphometric analysis

**Fig 3:**
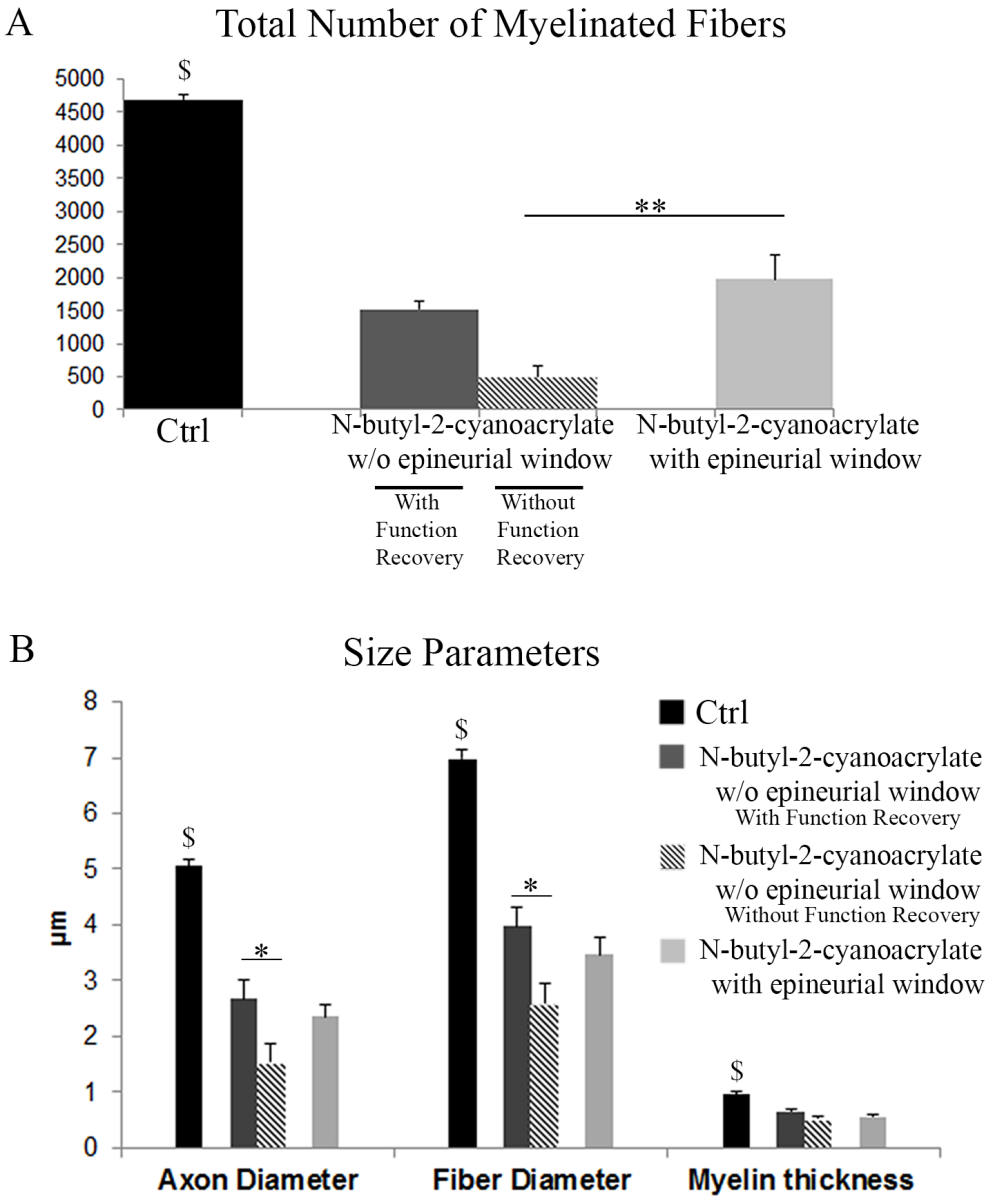
Histograms showing the results of stereological and morphometric evaluations. Data of Group 1 (N-butyl-2-cyanoacrylate w/o epineurial window) are divided into two parts: animal which displayed functional recovery (n = 3) and animals which did not (n = 4). Group 2 (N-butyl-2-cyanoacrylate with epineurial window) shows more myelinated fibres compared to animals of Group 1 (N-butyl-2-cyanoacrylate w/o epineurial window) without functional recovery. Significant differences are detectable for the analyzed size parameters between animals of Group 1_with functional recovery and animals of Group 1_without functional recovery. Values in the graphics are expressed as mean+standard error. $: p ≤0.001 between control and both the experimental groups; **: p≤0.01; *: p≤0.05.

Quantitative stereological evaluations for axon numbers, and morphometrical analysis for axon and fibre diameter and thickness of the myelin sheath, were performed in the distal part of the median nerve in both experimental groups, and compared to control values (Fig 3).

Also in this case we divided the results of Group 1 (N-butyl-2-cyanoacrylate w/o epineurial window) into two parts: animal which displayed functional recovery (n = 3) and animals which did not (n = 4).

Distal to the end-to-side coaptation, the number of myelinated axons was significantly decreased (p≤0.01) in both experimental groups (Group 1, both conditions and Group 2) compared to control nerve. Moreover, Group 1_without functional recovery showed significantly less (p≤0.01) myelinated fibre number compared to Group 2. Intriguingly, no significant differences were present between Group 1_with functional recovery and Group 2 (Fig 3A).

With regard to size parameters, both experimental groups (Group 1, both conditions and Group 2) showed smaller axon and fibre diameters compared to control (p≤0.01). Moreover, Group 1_without functional recovery, showed significantly smaller (p≤0.05) axon and fibre diameters compared to Group 1_ with functional recovery. No significant differences were present between Group 1_with functional recovery and Group 2. Finally, also myelin thickness was decreased after end-to-side repair compared to control, but the two experimental groups showed comparable values (Fig 3B).

## Discussion

### 1) Why nerve fibers sprout throughout end-to-side coaptation sites?

Several studies have investigated the starting mechanism of end-to-side nerve repair; in their accurate and original review, Bontioti and Dahlin [57] proposed three basic mechanisms consisting in a) contamination from axons regenerating from the proximal stump of the recipient nerve, b) true collateral sprouting from healthy fibres of the donor nerve and c) true axonal regeneration from damaged fibres of the donor nerve (“terminal sprouting”).

a) Contamination can be a problem and must be taken into account, especially when the site of end-to-side coaptation is near the site of recipient nerve lesion: traditional technique of treatment of neuroma can help.

b) Collateral sprouting from healthy fibres does not need donor nerve trauma: simple coaptation with the chemical call from the degenerated distal stump and from the distal effector is capable to induce axonal ingrowth from the donor trunk’s healthy fibres [58,59,60].

The origin of the axons has been demonstrated with double label techniques from Ranvier’s nodes proximal to coaptation site [61], but the level has been questioned, with some authors believing the closest Ranvier nodes to be involved [62] and other papers [11,63] claiming for a role played by more proximal structures. Maybe these different opinions have been produced by different techniques and methods of investigation, as just noted [64]: the former study [62] had been conducted on an epineurial window model (see below c) and using electrophysiological registration, and the latter by simple suture (see below c) to the donor nerve and double retrograde labelling technique [11] or epineurial window ad suturing (see below c), microtearing and histomorphometric analysis [63]. A role of interneuronal signalling in dorsal root ganglia had been claimed [65] and the hypothesis of a Central Nervous System origin has been recently regained [64].

Moreover, the efficacy of simple coaptation has been questioned [57,60] as far as it concerns axon number, myelination and functional results and other concerns have been introduced regarding the influence of axons ingrowing into the recipient nerve, whether motor [66,67,68,69,70], sensory [42,71,72] or even autonomic [73] in this kind of regeneration. Motor axons seem to need injury to start regeneration, whereas sensory axons can sprout spontaneously [42,57,72,74,75,76]. Finally, neuronal plasticity from pruning, including also assessment of agonistic donors to be selected [38,77,78] to brain involvement has also been investigated [57,79,80,81,82].

c) Trauma to the donor nerve is the cause of the third mechanism evoked by Bontioti and Dahlin [57]: terminal sprouting can be produced either opening a window in the trunk connective, or passing classical suture stitches.

As regards opening a window, several papers claim a window to be opened in the donor trunk; in particular, some clinical studies [38] show that a more complex and harder connective structure envelopes the nerve trunks and an epiperineurial window is needed to start end-to-side axonal regeneration; these data have confirmed previous experiences in rats and rabbits respectively [83,84]. On the other hand, in rats, a simple epineurial window seems enough [85,86,87]. A variant opening a larger epineurial window has also been proposed by Yan et al [88,89].

As regards suture without window, several authors suggest that coaptation without a window is capable to attract axons from the intact donor nerve trunk [1,4,6,11]. Interestingly, some authors [90] described a model of end-to-side coaptation without any window but with perineurial suture as more effective in producing axon regeneration than the same model with epineurial suture. Kelly et al [59] have also risen the question for a role played by pressure produced by the sutures as well as associated bleeding and inflammation.

The most popular technique, however, consists in passing stitches after opening an epineurial or an epiperineurial window (that is, coupling the two modalities of donor nerve trauma above mentioned) and results with this last technique are recognized by most authors as the best as far as it concerns number and myelination of axons and functional efficaciousness [10,14,59,72,80,91,92,93,94,95,96,97].

### 2) Glues in nerve reconstruction

Glues deserve separate considerations; in basic experiences, fibrin glue has been shown to be a good sealant in end-to-end nerve repair [98,99,100], and starting from Palazzi’s data [98], it has also been investigated as an interesting conduit for nerve regeneration, but its role has been questioned as not effective in end-to-side nerve regeneration [5]; however, these data have been questioned and fibrin’s role reconsidered [101,102].

Introduction of cyanoacrylates in both experimental and clinical practice has stimulated researchers’ curiosity, and has been applied to end-to-side nerve repair, but with debatable outcomes, whether questioned [43,48] or not [44,45,46,47]. Some recent experiences [103] are based on the debatable premise that cyanoacrylate produce inflammation and scar in the coaptation site.

#### a) Why end-to-side coaptation with glue?

All the experiences with glues have reported coupling gluing to opening an epineurial window. Indeed, even if some observations and hypotheses coming from basic sciences have been coupled with all these technique, no data but morphological analyses have been added to the biology of end-to-side nerve repair.

In fact, the role of Schwann cells and chemical signals (growth factors, cytokines from the distal stump products of Wallerian degeneration and reorganization, and also exocytosis products from the distal effector) has been suggested in attracting axons through a nerve injury or a gap [60], but not yet investigated in case of pure end-to side coaptation.

Both functional and morphological outcomes in our study confirm that end-to-side repair is followed by axonal regeneration in each group of animals, showing that axonal regeneration as well as myelination occurs both after opening an epineurial window and after simple coaptation of the distal stump of the cut nerve to the trunk of the healthy donor nerve. That is, from a qualitative point of view speaking, the event “regeneration” occurs whether the epineurium is disrupted or not. These data confirm the previous hypothesis from Viterbo [1] and Lundborg [6] that an important call comes from the distal stump to attract axons and can be explained with recent data from *in vitro* and experimental studies, as this same role speculated in case of end-to-end and/or graft or tubule repair [60] could be applied to our model.

Our study, however, demonstrates a marked and statistically significant difference between end-to-side axonal regeneration with epineurial window with respect to the group without window; indeed, not all the animals in the group without window showed functional recovery, whereas all the animals in the group with window recovered; this was also reflected from a morphological and morphoquantitative point of view, where the epineurial window spread out more axons and fibres. These data confirm previous evidences [10,14,59,72,80,91,92,93,94,95,96,97] that a trauma such as opening a window in the nerve trunk connective and passing a suture stitch stimulates axonal growth into the distal stump.

#### b) Is gluing with cyanoacrylates safe and does it produce nerve regeneration in the end-to-side model?

Our study shows that gentle applying of small quantities of cyanoacrylates on the coaptation site is enough to produce regeneration through this site, according to other experiences [44,45,46,47]. We did not experience either toxic local effects nor scar impeding regeneration, reported in other papers [43,48]. Indeed, neither fibrous tissue reaction nor other harmful effects on axonal regeneration were observed from a histologic evaluation. Dealing with toxicity through blood-nerve barrier, cyanoacrylate has recently been used in a rat model in a specific nanoparticle form to vehicle peptides into brain targets; no toxicity neither inflammation have been shown [104].

In our opinion, safe gluing with cyanoacrylates can be simply and carefully obtained and there is no need for isolating the coaptation site with a biological chamber [103], nor the stimulus by suture stitches is needed to obtain regeneration through end-to-side coaptation [90]. In this last case, axons sprouting occurs in a sutureless model, and our results demonstrate that it is detectable, even if it looks poorer, with a simple coaptation, but more evident and significant when coaptation is coupled to opening a window in the donor trunk, even if with the “escape” effect [36,38,39,40,41,42] that is with a reduction of axons, fibres and myelination in the donor trunk. This important side effect seems to occur whatever the coaptation method after opening a window in the nerve connective, even that it has been questioned by some important clinical papers [94,105], it still represents to date a major concern for clinical application of end-to-side coaptation [42,72].

## Conclusions

We can conclude that although regeneration *per se* applies to end-to-side repair after sutureless coaptation, an epineurial window is needed to achieve a significant number and quality of myelinated fibres as well as effective functional recovery. The use of cyanoacrylate glue provides to the microsurgeon a valid alternative to suturing for end-to-side nerve coaptation.
